# Applications of Proteomic Tools to Study Insect Vector–Plant Virus Interactions

**DOI:** 10.3390/life10080143

**Published:** 2020-08-07

**Authors:** Priyanka Mittapelly, Swapna Priya Rajarapu

**Affiliations:** 1 Department of Entomology, Ohio Agricultural Research and Development Center, The Ohio State University, Wooster, OH 44691, USA; priyanka.mittapelly@usda.gov; 2 USDA APHIS PPQ, 5936 Ford Ct, Ste. 200, Brighton, MI 48116, USA; 3 Department of Entomology and Plant Pathology, North Carolina State University, Raleigh, NC 27695, USA

**Keywords:** insect vectors, plant viruses, vector–virus interactions, proteomic tools, applications

## Abstract

Proteins are crucial players of biological interactions within and between the organisms and thus it is important to understand the role of proteins in successful partnerships, such as insect vectors and their plant viruses. Proteomic approaches have identified several proteins at the interface of virus acquisition and transmission by their insect vectors which could be potential molecular targets for sustainable pest and viral disease management strategies. Here we review the proteomic techniques used to study the interactions of insect vector and plant virus. Our review will focus on the techniques available to identify the infection, global changes at the proteome level in insect vectors, and protein-protein interactions of insect vectors and plant viruses. Furthermore, we also review the integration of other techniques with proteomics and the available bioinformatic tools to analyze the proteomic data.

## 1. Introduction

**Figure 1:**
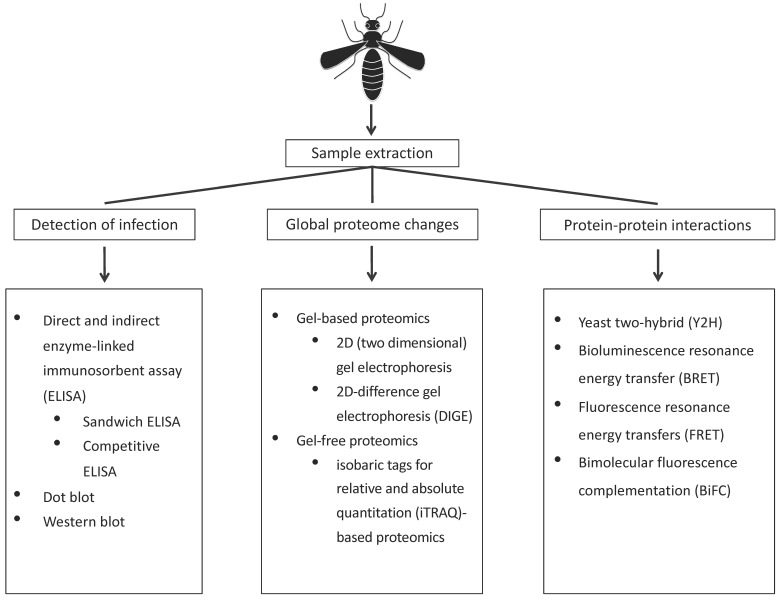
Summary of proteomic techniques to study different stages of insect vector-virus interactions, including detection of infection, global proteome changes and protein-protein interactions.

Insects are the largest class of plant virus vectors with a significant economic impact [1]. Although over the last decade next generation sequencing tools have been extensively used to study insect vector and plant virus interactions, proteomics has significantly contributed in identifying the proteins that are crucial for these interactions. Various technological advances in proteomics are further allowing progress in the area of insect vector–virus interactions both at organismal and cellular levels. Insect vectors and plant viruses have various phases of interactions. Viruses from an infected plant are acquired by the insect vector (acquisition phase), retained in its tissues (retention phase) and transmitted (transmission phase) to a virus-free plant (inoculation phase). Based on the virus acquisition, inoculation and retention time by the vector, three transmission strategies have been identified, including non-persistent, semi-persistent and persistent [2,3,4]. Non-persistent plant viruses are usually retained in the insect stylet [5,6]. Semi-persistent plant viruses are taken up by the insect vector, bind to the chitin layer and do not pass beyond the foregut [7,8,9]. On the other hand, persistent viruses are retained in different insect tissues and invade the salivary glands, to readily transmit the virus to a new host while feeding [10]. There are two sub-classes of persistent viruses, propagative and non-propagative viruses. Propagative viruses are known to replicate within the insect host whereas non-propagative viruses do not replicate within the insect host [11,12,13,14]. Numerous studies to date have reviewed these complex interactions of plant viruses with their insect vectors [10,12,15,16,17]. Several proteins within these complex interactions have been identified by both conventional and contemporary proteomic tools. In this review, we summarize the available proteomic techniques to study the highly coevolved insect vector–plant virus interactions. An overview of various proteomics techniques used at specific stages of interactions, including detection of infection, identification of global proteome changes, and protein-protein interactions are described in the next sections (Figure 1).

## 2. Proteomic Approaches to Study of Vector–Virus Interactions

### 2.1. Detection of Infection

Although there are various techniques currently being used for detection of viruses, the conventional method, enzyme-linked immunosorbent assay (ELISA), has been popular since its development in 1977 [18], and has been extremely useful to scientists in various fields of animal–virus interactions [18,19,20]. The basic principle of ELISA is to capture the target antigen (or antibody) in the samples by using epitope- (binding site on antigen) specific antibodies cross-linked to an enzyme that converts the substrate to its respective product, which is colorimetrically measured to quantify the antigen (or antigens). Thus, by enzyme substrate reaction, the target molecules can be detected and quantified [21]. The ELISA approach can be grouped into four main categories, including direct ELISA, indirect ELISA, sandwich ELISA and competitive ELISA. Direct ELISA uses only one antibody to detect a single epitope on the viral antigen whereas indirect ELISA uses two antibodies, including a primary antibody that binds to the epitope and an enzyme-linked secondary antibody that binds to the primary antibody (double antibody sandwich ELISA, DAS-ELISA) [21]. Another type of indirect sandwich ELISA is a triple antibody sandwich ELISA (TAS-ELISA) which involves antibodies from two different species of organisms. Although faster and economical, these approaches produce background noise. On the other hand, sandwich ELISA is relatively more sensitive and robust as two antibodies bind to two non-overlapping epitopes on the antigen. Any of the above ELISA approaches can be adapted to competitive ELISA, where a competition between a reference antigen and sample antigen to the primary antibody is used to leverage the abundance of antigen of interest in the sample [21].

The major advantage of ELISA is that it can be used to test a large number of samples in a relatively short period of time. This technique was extensively used in the detection and diagnosis of many plant viruses in various insect vectors (Table 1). On the other hand, the major disadvantages include low flexibility, antibody instability and cross-reactivity from a secondary antibody. Technological advances are, however, providing improved ELISA or ready-to-use test kits to overcome the disadvantages. In addition to ELISA, dot blots and western blots have also been used to detect infection, which primarily include an antibody-based detection of the virus in tissue lysates that are either directly blotted or separated and transferred from a protein gel onto a nitrocellulose membrane [22].

### 2.2. Techniques to Identify Global Changes to the Vector Proteome upon Infection

Studying the global proteomic responses of an insect vector enables an understanding of both tissue and organismal changes incurred by the virus infection. Most of the global proteome studies focused on the vectors of persistent, propagative and non-propagative viruses. These groups of viruses navigate within the host environment and require sophisticated mechanisms to overcome the host cellular and molecular barriers, such as insect immune system. Techniques available for global proteomic studies can be broadly classified into gel-based, where samples are separated on a polyacrylamide gel before identification, and gel-free proteomic techniques where proteins are identified directly from the complex mixture by spectrometry techniques discussed below. Both these techniques have been used to identify differentially expressed proteins in vectors infected with plant viruses. The principles and experimental details of these techniques have been discussed in detail elsewhere and hence are focused briefly in this review [36,37,38].

#### 2.2.1. Mass Spectrometry for Global Proteomics

Mass spectrometry has revolutionized proteomics by facilitating a high throughput identification of proteins. Simple to complex mixtures of proteins are separated and identified by liquid chromatography-mass spectrometry (LC-MS), wherein the separated proteins are ionized by various methods, such as electrospray ionization and matrix-associated desorption ionization, both of which ionize the analytes from liquid to gaseous phase. Working principles and application of mass spectrometry are well-studied and have been reviewed extensively (example, [38,39]). Prior to separation by LC-MS, proteins are digested to peptides by trypsin and separated on a column in the presence of a mobile solvent. The separated peptides are sequentially ionized (MS/MS) to precursor and their fragment ions, which improves the resolution to identify amino acids in the peptides. Mass of the precursor ions is used to screen the database of peptides and their fragmentation pattern generated in silico from a transcriptome or genome database for data-dependent acquisition of protein sequences. Matching peptides of the spectrum are chosen based on the highest scoring peptide, depending on the scoring algorithms used [40,41]. This also highlights one of the major limitations for proteomics as the availability of sequence databases is vital to identify proteins in insect vectors and most of the insect vector genomes are yet to be sequenced.

#### 2.2.2. Sample Collection and Protein Isolation for Global Proteome

Irrespective of the proteomic approach, efficient extraction of proteins along with maintaining the integrity of the proteins is important for good quality data. Studies conducted in insect vectors have so far applied various tissue collection strategies, depending on the tissue of interest. For whole organismal proteomics, individuals were either collected in microcentrifuge tubes, flash frozen in liquid nitrogen and stored at −80 °C until protein extraction [42], or proceeded directly for protein isolation in the lysis buffer [43]. With respect to tissue level proteomics, dissected tissues were processed in a lysis buffer [44]. Lysis buffer used for protein isolation from insect tissues contained a protein denaturing agent, such as urea and dithiothreitol (DTT) along with detergents, such as 3,3-cholamidopropyl dimethylammoniol-1-propanesulfonate (CHAPS) and sodium dodecyl sulfate (SDS) to disrupt the membranes for an efficient release of proteins. For the two-dimensional gel electrophoresis (2D gel) approach, the lysis buffer also includes an ampholyte to facilitate separation of proteins on a 2D gel. However, buffers used for dissection and tissue collection have not been well-described for insect vectors of plant viruses. Based on the knowledge from other insect tissue collections, different dissection buffers used included 1X phosphate buffer saline (pH 7.00, [45,46,47]) with or without protease inhibitors, ringer solution (123 mM NaCl, 1.5 mM CaCl₂, 5 mM KCl, [48,49]) and lysis buffer (9.5 M urea, 2% CHAPS, pH 3–10 and 1% dithiothreitol (DTT), [50]). For certain insect vectors, such as aphids, specific protein extraction methods were optimized [51].

#### 2.2.3. Gel-Based Proteomics

For gel-based proteomics, extracted proteins were separated on a polyacrylamide gel based on isoelectric point (pH at which the net charge of the protein is 0) and molecular weight. Typically, 10,000 proteins can be separated by 2D gel electrophoresis [37,38]. For quantitative 2D-difference gel electrophoresis (2D-DIGE), samples are usually labeled with fluorescent dyes, such as 1-(5-carboxypentyl)-1′-propylindocarbocyanine halide (Cy3) *N*-hydroxysuccinimidyl ester and 1-(5-carboxypentyl)-1′-methylindodicarbocyanine halide (Cy5) *N*-hydroxysuccinimidyl ester. Labeled samples are pooled and separated to identify different fluorophores. This technique was used to identify differentially expressed proteins between cereal yellow dwarf virus (CYDV)-RPV transmission-competent and refractive aphid populations to identify biomarkers for virus transmission [52]. This study highlighted the difference in the protein properties, such as isoelectric focusing of symbiont proteins and also functional differences in the two aphid populations. Heritability of the proteins involved in virus transmission are compared by comparing the proteins from parental and F2 populations, expanding the scope of proteomics to hereditary studies particularly in the case of virus transmission. Similarly, another study identified proteins expressed in the first instar larvae of western flower thrips, which is a crucial stage for virus acquisition [42]. This study has identified proteins involved mostly in immunity, including tubulin alpha-1 chain, beta tubulin, glutaredoxin 5, heat shock protein, cysteine protease and lethal giant larvae homologue as differentially expressed in the infected larvae.

#### 2.2.4. Gel-Free Proteomics

In contrast to gel-based proteomics, gel-free proteomics offers an advantage of minimum sample requirements, nanograms compared to micrograms, which overcomes the challenge to studying small insect vectors, which are also among the most competent vectors of plant viruses. Gel-free proteomic techniques can be carried out as either labeled or label-free quantitative proteomics. Labeled proteomic approaches enable the evaluation of a mixture of samples in one run which can later be identified by the presence of a unique label of the samples. Samples are typically labeled with radioisotopes via metabolic labeling, a process feasible only for cell cultures which was soon replaced by chemical modification of specific amino acids in intact proteins, such as an isotope-coded affinity tag [53], isotope-coded protein label [54] and the most advanced being the isobaric tags for relative and absolute quantitation (iTRAQ) [55]. Up to four samples can be compared with the reagents available for iTRAQ. The advantage of this approach to studying insect vectors has been highlighted by the tissue-specific proteomic studies of small brown planthopper, aphids and whiteflies [43,44,56]. Differential proteomics of small brown planthopper ova infected with rice stripe virus RSV relative to uninfected tissue identified 147 differentially expressed proteins between the two tissues, with proteins involved in ribosome upregulated and primary metabolic processes downregulated [44]. However, only 334 proteins were identified from the ova, which could attest to its specialized function. Another study using iTRAQ identified the proteomic responses of whiteflies with various begomoviruses, tomato yellow leaf curl virus (TYLCV) and papaya leaf curl China virus (PaLCuCNV) [56]. From this study, a total of 3555 proteins were identified from all the treatments with 259 for TYLCV and 395 for PaLCuCNV differentially expressed proteins in virus infected relative to uninfected insects [44]. These two studies provide an example of the number of proteins that can be identified in insect vectors based on the sample source. In another study, label-free proteomics of 500 aphid stylets (~267 µm) identified 15 unique cuticle proteins out of 141 total proteins, demonstrating the potential of label-free proteomics to identify proteins from small samples, such as aphid stylets [57]. With the availability of these tools, it is possible to study tissue-specific proteomics of insect vectors.

### 2.3. Techniques to Identify Vector Proteins Interacting with Virus Proteins

Successful progression of virus transmission occurs with coordinated protein-protein interactions both at the tissue and cell levels. Existing proteomic techniques to study these interactions can be classified into in vitro, in vivo and in silico [58]. Techniques within in vitro studies include affinity chromatography, tandem affinity purification, coimmunoprecipitation, phage display, protein arrays, protein fragment complementation, X-ray crystallography and NMR spectroscopy (reviewed in [58]). Yeast two-hybrid (Y2H) and bioluminescence resonance energy transfer (BRET), fluorescence resonance energy transfers (FRET) and bimolecular fluorescence complementation (BiFC) are some of the in vivo techniques used to study protein-protein interactions. Advancements in bioinformatics have enabled the development of in silico protein-protein interaction tools to overcome the time limitations of identifying specific interactions. Currently, this approach is favorable, with the rapid increase in next generation sequencing techniques generating gigabytes of data. There are several softwares available to determine protein-protein interactions in silico [58]. Nevertheless, it is not a standalone approach and the bioinformatic predictions need to be validated in vitro or in vivo.

The most common in vitro assays used to study insect vector–plant virus interactions include 2D gel overlays, where the vector proteins are separated on 2D gels, transferred onto the membrane and blotted with purified virus particles. The virus particles on the membrane are identified by antibodies against virus particles via far Western blotting. An overlay of the gel and the blot identifies the vector proteins that are bound to the virus particles. These proteins are further identified by the nano LC-MS/MS. For example, this approach has identified green peach aphid, *Myzus persicae* proteins, including the receptor for activated C kinase, actin and glyceraldehyde-3-phosphate dehydrogenase interacting with beet western yellow virus [59]. Similarly, another study in western flower thrips identified six proteins, including three cuticle-associated proteins, a cyclophilin, enolase and mATPase interacting with tomato spotted wilt virus (TSWV) [60]. These interactions are further confirmed by in vivo techniques, including split-ubiquitin membrane-based Y2H and colocalization. A similar approach in whiteflies identified cyclophilin as an interactor of TYLCV [61]. Cyclophilin is the most abundant multifunctional protein with roles in protein folding and trafficking, cell signaling, and it also plays a role during viral infection across many organisms [60,62,63,64]. Moreover, it has been observed in abundant quantities in insect midgut and salivary glands [65].

Most of the vector proteins that interact with viral proteins have been identified by Y2H. The principle behind this technique is based on the interaction between yeast-specific DNA binding protein (bait) and transcription activation protein (prey), which in proximity transcribe a reporter gene. The proteins of interest are fused with either of these two yeast-derived proteins in yeast expression vectors. These vectors are cotransformed into yeast and, upon interaction of the candidate proteins, the reporter gene is expressed, and positive colonies are identified and sequenced to determine the bait proteins interacting with a prey protein. Using this approach, 66 brown planthopper proteins interacting with the RSV pc3 protein have been identified. These results were further confirmed by chemiluminescent coimmunoprecipitation assay and glutathione S-transferase (GST) pull down assays [66]. A similar study in brown planthopper identified RPL18 interacting with RSV N protein [67]. In white-backed planthopper, *Sogotella furcifera*, 24 vector proteins interacting with the non-structural protein, P7-1, of southern rice black-streaked dwarf virus (SRBSDV) were identified via Y2H and further confirmed with coimmunoprecipitation [68]. Yet in another study, direct interaction of a green peach aphid, cuticular protein with CMV capsid protein, was determined with Y2H [69]. Y2H was also used to confirm the results identified by in vitro studies, such as comparative proteomics of the bird cherry oat aphid, *Rhopalosiphum padi*, and the green bug aphid, *Schizaphis graminum*, infected with barley yellow dwarf virus-GPV (BYDV-GPV) using iTRAQ. Validation of iTRAQ results with Y2H confirmed that 25 and eight vector proteins interacted with the virus read through domain and the coat protein, respectively, further validating the global proteomic approach. Most of the proteins identified as interacting with virus proteins involved in primary energy metabolism, synaptic vesicle cycle, the proteasome pathway and the cell cytoskeleton organization pathway [43]. Advantages and disadvantages of these techniques along with the insect vector in which the technique was used are described in Table 2. A limitation of any of these techniques to study insect vectors is the relative body size of the vectors, and thus a large quantity of insect tissue is required for these approaches.

## 3. Combining Proteomics with Multiple ‘-Omics’-Based Techniques

Proteomics alone provides a greater perspective of understanding the complexity of vector–virus interactions. However, combining proteomics with other “-omics” techniques, such as transcriptomics and functional genomics tools, complement each other and synergize for a deeper understanding of insect vector–virus interactions. In addition, studies have combined genetic and proteomic approaches to identify greenbug aphid proteins (including luciferase and cyclophilin) associated with CYDV-RPV transmission [81,82,83]. In another study on the Asian citrus psyllid that transmits bacteria, *Candidatus Liberibacter asiaticus* combined transcriptomics and proteomics (mass spectrometry) with fluorescent in situ hybridization (FISH) to understand the interactions between host and pathogen at the tissue level [84]. Using RNA sequencing, 83 non-coding RNAs were identified as the most abundant transcripts in the transcriptome and proteomics has helped in determining the protein level changes in bacterial symbiont (*Wolbachia*), when the pathogen is present. Adding microscopic studies, such as FISH, confirmed that *Wolbachia* cells are more prevalent in gut cells of the infected Asian citrus psyllid, *Diaphorina citri*, providing more insights on host–pathogen interactions [76]. On the other hand, studies have integrated different proteomic techniques to identify proteins that interact with the plant viruses [43]. A study on BYDV-GPV transmitted by insect vectors bird cherry oat and greenbug aphids identified 33 proteins using the iTRAQ method and 28 proteins using the Y2H system with only two proteins overlapping between both techniques, indicating their independent potential [43]. A protein interaction network of the identified proteins was predicted using STRING software, and the biological functions of the proteins identified by both techniques were complementary. Similarly, combining Y2H and functional genomics tools, such as RNAi, are powerful in determining the functional role of the protein in virus transmission [80].

Future studies on virus–vector interactions using multiple -omic levels, including genomics, transcriptomics, proteomics and metabolomics can provide unique resources on how viruses affect key processes in their respective hosts. In addition to ‘-omics’, the experimental methods should be further extended to protein-protein interactions, post-translational protein modifications and epigenetics [85]. Integrating these approaches can provide specific and global information on vector–virus interactions (on either perspective) to help identify proteins that can be used for targeted disruption.

## 4. Bioinformatic Analysis of Proteomics Data

Proteomic output either from global or targeted proteomes comprise a list of proteins identified by programs such as SEQUEST [40] and MASCOT [41]. An annotated transcriptome or genome reference used to identify the peptides from mass spectrometry overcomes the protein annotation step. However, in the case of insect vectors, more than 50% of the transcriptomes remain unannotated. With the availability of new sequences deposited to public databases, such as NCBI, it is possible to acquire updated annotations for insect vector proteomes. The Basic Local Sequence Alignment (BLAST) application allows identification of homologous proteins in protein databases, such as non-redundant (nr) databases and UniProt–Swiss-Prot databases. These analyses can be performed on user-friendly web platforms or on a local computer command prompt or Unix terminals, depending on the operating system.

Annotated proteins can be assigned a putative function as described by the “Gene Ontology” (GO) resource (http://geneontology.org/), which hierarchically clusters proteins into three GO terms, biological process, cellular component and molecular function. Although it is feasible to obtain gene ontology terms based on the gene or protein ID from the gene ontology website, it is best to use platforms that can assign GOs for insect vector proteins. Platforms, such as Blast2GO [86], can assign GO terms to all the annotated proteins. Alternative platforms, such as DAVID [87,88] and PANTHER [89], can assign GO terms to the proteins in a given gene or protein list. However, it is important to remember that DAVID and PANTHER work efficiently for model organisms. Proteins can also be designated to biological pathways by the Kyoto Encyclopedia of Genes and Genomes (KEGG) database. Platforms, such as BlastKOALA [90], can be used for pathway annotation. Other molecular features of the proteins, such as presence of signal peptide, cellular localization and transmembrane domain can be predicted by the softwares, including SignalP [91,92], DeepLoc [92] and TMHMM [93] respectively.

Similar to transcriptomics, differentially expressed proteins can be identified from the shotgun quantitative proteomic techniques, such as iTRAQ or label-free proteomics. Unlike transcriptomics, preprocessing of protein abundances requires accounting for missing data since different peptides of the same protein would be identified across different samples (reviewed in [94]). Most of the R packages identifying differentially expressed proteins includes data preprocessing steps. Some of the approaches for identifying differentially expressed proteins include Student’s *t*-test, Limma [95], Differential Enrichment analysis of Proteomic data (DEP) [96] and MSstats [97].

Another bioinformatic approach for systems biology understanding is performing network analysis to identify coregulated proteins. Weighted gene correlation network analysis, WGCNA, is popularly used in transcriptomics, but can also be applied to identify coregulated proteins [98,99]. Input for performing network analysis is the missing data imputed and normalized protein abundance data. Proteins are grouped into modules based on the similarity of the expression across all the samples. Alternatively, putative protein-protein interactions can be identified within the differentially expressed genes by the STRING program, a functional protein association networks application [100]. The STRING database contains 5090 organisms and more than 2000 protein interactions. An interaction network of the input proteins is constructed based on the interaction database. Although this database has an exhaustive list of organisms, *Acyrthosiphon pisum*, pea aphid was the only insect vector of plant viruses that made it to the list.

## 5. Conclusions and Future Directions

In this era of functional genomics, there has been an exponential growth in the use of proteomic tools and most of these were mainly applied to biomedical-, human- and plant-related research. The studies on insect vector–plant virus interactions that take advantage of proteomic tools are scarce or just evolving. Proteomics is a comprehensive tool that can be used for protein identification, quantification and possible modifications at the tissue and cellular levels. There are several techniques available to study protein-protein interactions, including tandem affinity and affinity purification mass chromatography, coimmunoprecipitation, phage display, protein microarrays, protein fragment complementation, chemical cross-linking and BioID to study insect vector and plant virus protein-protein interactions.

**Figure 2:**
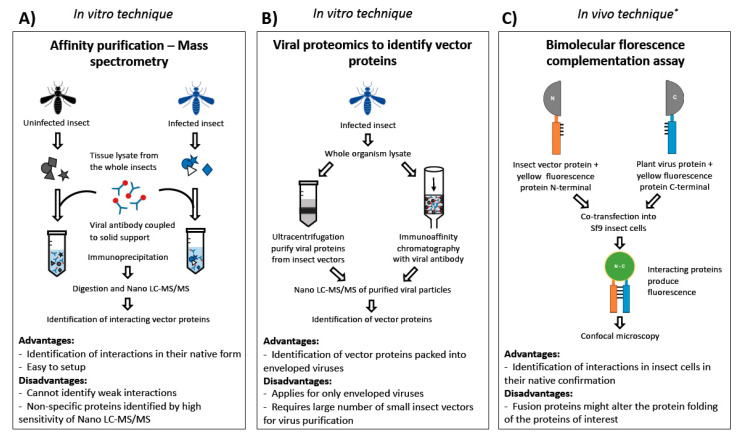
Future approaches to identify insect vector proteins interacting with the plant virus proteins. (**A**) Affinity purification mass spectrometry to identify the vector proteins interacting with viral proteins in their native form by nano liquid chromatography-tandem mass spectrometry (LC-MS/MS). Viral proteins are immobilized onto a solid support and vector proteins are immunoprecipitated and identified by mass spectrometry. (**B**) Viral proteomics to identify vector proteins packed into the viral particle. Viral proteins from the infected insect vector are separated either by ultracentrifugation or immunoaffinity chromatography and vector proteins are identified by mass spectrometry. (**C**) Bimolecular fluorescence complementation assay to identify interacting proteins in vivo. Viral and vector proteins of interest are fused to N and C terminals, respectively, and positive interactions lead to fluorescence. * Proteins identified in vitro could be validated using the in vivo techniques.

Some of these techniques have been applied to study plants and their respective virus interactions. For example, protein interactions between TYLCV and *Nicotiana benthamiana* were studied using affinity purification mass spectrometry analysis. In this study, the viral GFP fusion proteins expressed in the plants were purified using anti-GFP antibody-coated columns to separate proteins bound to viral GFP fusions [101]. Virus-interacting proteins were visualized on polyacrylamide gels and identified by mass spectrometry. A similar approach can be applied to study interacting proteins of insect vectors with viral proteins (Figure 2A). However, obtaining sufficient amounts of protein samples from some smaller insect vectors becomes one of the limitations for implementing such technology. However, with smaller quantities of proteins required for label-free proteomics affinity purification mass spectrometry might soon be possible in insect vectors. Proteomics of viral particles purified from a vector could provide information about host proteins packed into viral particles, particularly in enveloped viruses [102]. With the aid of chromatographic approaches, such as immunoaffinity chromatography [103], purifying viral particles from insect vectors could overcome the limitation of the number of insects required (Figure 2B).

For a more targeted approach, peptide arrays can be performed to identify specific protein interactors within a protein family. For example, Webster et al. [77] have performed peptide arrays with aphid cuticular proteins having Rebers and Riddiford domains to identify the consensus peptide sequence specifically binding to P2 helper components of CMV particles. For vectors with small body size, in vitro binding assays with viral GFP fusion proteins can be performed to identify the spatial interactions of viral proteins with insect structures [102]. Such assays offer an advantage to study the interactions in non-persistent viruses where the virus interacts with the insect mouth parts. The most advanced technique used so far to study the insect–virus interactions include chemical cross-linking coupled with mass spectrometry, wherein the interacting proteins are covalently linked using a cross-linker. The linker reacts with amino acid side chains of two interacting proteins and provides information on the topology of the protein-protein interactions. Aphid proteins interacting with the virus proteins were identified with chemical cross-linking technology [76]. Proximity-dependent labeling techniques have been widely used to study protein-protein interactions in a wide range of systems other than insects (reviewed in [104,105]). The underlying principle of this technique is that bait protein fused with an enzyme chemically tags the interacting proteins with biotin, which are later purified by capturing on a streptavidin affinity matrix and identified by LC-MS/MS. Several enzyme fusions have been developed for this assay, including biotin ligase, ascorbate peroxidase and horseradish peroxidase, which tag the interacting proteins either with biotin or biotin derivatives to the interacting proteins. However, all these in vitro techniques require in vivo confirmation for which BiFC is an ideal approach as the protein interactions can be tested in insect cell lines. The basic principle of this approach is that proteins of interest are fused to a yellow fluorescent protein, and in the event of interaction, fluorescence is observed [106] (Figure 2C). Although the application of proteomic techniques to study insect vector–plant virus interactions is in its infancy, the availability and accessibility of these techniques along with cross-disciplinary collaborations will enable advancements in the field of insect vector–plant virus interactions.

## Figures and Tables

**Table 1:** In vitro detection of virions using the conventional method: enzyme-linked immunosorbent assay (ELISA) in insect vectors.

Insect Vector	Virus	Antigen	ELISA Type	Reference
*Rhopalosiphum padi* and *Rhopalosiphum maidis*	Barley yellow dwarf virus (BYDV)	Antiserum made by injecting PAV and MAV (two isolates transmitted by *R. padi*) to rabbits	Double antibody sandwich (DAS) ELISA	Lister and Rochow 1979 [23]
*Aphis gossypii*	Plum pox potyvirus (PPV)-D and M serotypes	PPV-D- and PPV-M-specific monoclonal antibodies	Double antibody sandwich indirect (DASI) ELISA	Olmos et al. 1997 [24]
*Myzus persicae*	Beet yellow virus (BVY)	BVY-specific monoclonal antibody	Triple antibody sandwich (TAS) ELISA	Stevens et al. 1997 [25]
*Myzus persicae*	Alfa mosaic virus (AMV)	Glutaraldehyde-fixed AMV antisera raised in rabbits	Indirect double antibody sandwich (IDAS) ELISA	Ahoonmanesh et al. 1990 [26]
*Bemisia tabaci*	Lettuce infectious yellows virus (LIYV)	Coat protein (CPm)	Double and triple antibody sandwich (DAS and TAS) ELISA	Chen et al. 2011 [9]
*Peregrinus maidis*	Maize stripe virus (MStpV)	Purified non-capsid protein (Falk 1983)	Double antibody sandwich (DAS) ELISA	Falk et al. 1987 [27]
*Peregrinus maidis*	Maize stripe tenuivirus (MStV)	MStV-US * [28]	Indirect ELISA	Ammar et al. 1995 [29]
*Cicadulina chinai*	Maize yellow stripe virus (MYSV)	MYSV * [30]	Direct antigen coating (DAC) ELISA	Ammar et al. 2007 [31]
*Cicadulia mbila*	Maize streak virus (MSV)	MSV	Indirect double antibody sandwich (IDAS) ELISA	Reynaud and Peterschmitt 1992 [32]
*Frankliniella occidentalis*	Tomato spotted wilt virus (TSWV)	Nucleocapsid and glycoprotein (G_N_)	Triple antibody sandwich (TAS) ELISA	Margaria et al. 2014 [33]
*Frankliniella occidentalis*	Tomato zonate spot virus (TZSV)	TZSV *	Double antibody sandwich (DAS) ELISA	Yin et al. 2014 [34]
*Bemisia tabaci*	Tomato yellow leaf curl Thailand virus (TYLCTHV)	Recombinant coat protein (CP)	Triple antibody sandwich (TAS) ELISA	Seepiban et al. 2017 [35]

* Antiserum was used for detection in ELISA.

**Table 2:** In vitro and in vivo protein-protein interaction techniques used to identify insect vector proteins interacting with plant viruses.

Approaches to Study Protein-Protein Interactions	Pros/Cons	Examples of Insect Vector–Plant Virus System Using the Approach
***In Vitro Techniques***		
**One- or Two-Dimensional Virus Overlay/Far Western Blot Assays**	**Pros:** Detects interactions between native proteins. Does not always require antibodies if a tagged protein is available. Relatively fast qualitative approach. **Cons:** Non-specific interactions due to protein denaturation. Poor resolution of protein separation leads to missing interactions.	*Myzus persicae–*Potato leaf virus [70] *Frankliniella occidentalis*–Tomato spotted wilt virus [60,71,72] *Myzus persicae*–Beet western yellow virus [59] *Acyrthosiphon pisum*–Pea enation mosaic virus I [73] *Sitobion avenae*–Barley yellow dwarf virus-MAV [74,75] *Laodelphax striatellus*–Rice stripe virus [69]
**Coimmunoprecipitation**	**Pros:** Identify interactions from the tissue lysate in the native form of the protein. **Cons:** Only a single antibody targeting a specific antigen can be used. Low-affinity and transient interactions might not be detected.	*Sogatella furcifera*–Southern rice black-streaked dwarf virus [68]
**Cross-Linking/Protein Interaction Reporter Technology**	**Pros:** Identify protein interactions in the native confirmations.Multiple protein interactions can be determined. Transient or weak interactions can be identified. Can be adapted for in situ protein interactions. **Cons:** Cross-linkers can cause stearic hindrance for cross-linking interacting proteins. Significant sample loss due to multiple steps.	*Myzus persicae–*Potato leafroll virus [76]
**Protein or Peptide Arrays**	**Pros:** High throughput method. Can identify consensus protein sequences binding to the virus. **Cons:** Difficult to produce recombinant proteins. Cost and time required to generate the arrays.	*Aphis pisum–*Cucumber mosaic virus [77]
***In Vivo Techniques***		
**Yeast Two-Hybrid**	**Pros:** Interactions can be identified in living cells. Can be used to confirm the interactions observed in in vitro proteomic techniques. **Cons:** Protein structure may be altered due to fusion to the activation or DNA-binding domain of the transcription factor. Protein folding and post-translational modifications might be different than the insect vectors. Higher rates of false positives and false negatives.	*Bemisia tabaci–*Tomato yellow leaf curl sardinia virus [78] *Spodoptera furcifera–*Southern rice black-streaked dwarf virus [67] *Myzus persicae–*Cucumber mosaic virus [69] *Laodelphax striatellus*–Rice stripe virus [66,67,79] *Myzus persicae*–Turnip yellow virus [80] *Frankliniella occidentalis*–Tomato spotted wilt virus [60]
